# A pilot trial of an online guided self-help cognitive behavioral therapy program for bulimia nervosa and binge eating disorder in Japanese patients

**DOI:** 10.1186/s13030-023-00294-1

**Published:** 2023-11-10

**Authors:** Noriaki Ohsako, Hiroshi Kimura, Tasuku Hashimoto, Yutaka Hosoda, Yosuke Inaba, Masaomi Iyo, Michiko Nakazato

**Affiliations:** 1 Department of Psychiatry, Bellvitge University Hospital-IDIBELL, c/ Feixa Llarga s/n, L’Hospitalet de Llobregat, Barcelona 08907 Spain; 2 Department of Psychiatry, Chiba University Graduate School of Medicine, 1-8-1 Inohana, Chuo-ku, Chiba-City, Chiba 260-8670 Japan; 3 Department of Psychiatry, International University of Health and Welfare (IUHW), 852 Hatakeda, Narita-City, Chiba 286-8520 Japan; 4 Department of Psychiatry, Gakuji-Kai Kimura Hospital, 6-19 Higashihon-cho, Chuo-ku, Chiba-City, Chiba 260-0004 Japan; 5 Department of Psychiatry, Kisarazu Hospital, 2-3-1 Iwane, Kisarazu-City, Chiba 292-0061 Japan; 6 Clinical Research Center, Chiba University, 1-8-1 Inohana, Chuo-ku, Chiba-City, Chiba 260-8670 Japan

**Keywords:** Bulimia nervosa, Binge eating disorder, Cognitive behavioral therapy, Internet-based Guided Self-Help CBT (iGSH-CBT), Online therapy

## Abstract

**Background:**

The purpose of this study was to develop an internet-based Guided Self-Help CBT (iGSH-CBT) for Bulimia Nervosa (BN) / Binge Eating Disorder (BED) for Japanese patients and to test its feasibility.

**Methods:**

A single-arm feasibility study. After baseline assessment, patients underwent a 16-week iGSH-CBT program, our Japanese adaption of the European-based Salut BN program. During the treatment period, weekly email support from trained counselors was provided. Evaluations were performed at baseline, after 8 weeks, at the end of the 16-week intervention, and at 2 months after treatment had ended. The primary outcome measure was the change in the weekly frequency of objective binging. Secondary outcomes were the change in the weekly frequency of objective purge episodes, responses on self-report questionnaires of the frequencies of binging and purging, psychopathological characteristics of eating disorders found on BITE, EDE-Q, EDI-2, HADS and EQ-5D, measurements of motivation, and completion of intervention (vs. dropout).

**Results:**

Participants were 9 female outpatients with BN (*n* = 5) or BED (*n* = 4), of whom 8 (88.9%) attended the assessment at the end of the 16-week intervention. Mean age was 28 years (SD = 7.9). Percent change of the weekly frequency of objective binging was -4.40%, and at the end of the 16-week intervention 25% of the participants had achieved symptom abstinence.

**Conclusions:**

No adverse events were observed during the treatment period and follow-up, and the implementation and operation of the program could be performed without any major problems, confirming the feasibility of iGSH-CBT for BN and BED for Japanese patients. Although no significant change was observed in the weekly frequency of objective binging, the abstinence rate from bulimic behaviors of those who completed the assessments was 25.0% at the end of treatment, and the drop-out rate was 11.1%. iGSH-CBT may be an acceptable and possibly even a preferred method of CBT delivery for Japanese patients with BN or BED, and our Japanese adaptation of Salut BN seems feasible.

**Trial registration:**

UMIN, UMIN000031962. Registered 1 April 2018 - Retrospectively registered, https://center6.umin.ac.jp/cgi-open-bin/ctr/ctr_view.cgi?recptno=R000036334

## Background

Eating disorders are now frequently seen mental disorders, not only in western countries where they first became commonplace, but also in Asian countries, including Japan. Indeed, their pervasiveness in Japan is increasing and is associated with rapid cultural changes in relation to body image over the last three decades [1–4].

Bulimia Nervosa (BN) is an eating disorder that crucially impairs the mental and physical health as well as the social function of affected individuals [5]. A recent study indicated a lifetime prevalence of DSM-5-defined BN in a female population aged 18 years and older in the United States of 0.46%, and BED at 1.25% [6]. Patients with BN or BED often present with suicide ideation and attempts [7, 8]. A meta-analysis by Preti et al. based on 16 studies published between 1988 and 2009 demonstrated that BN patients in western countries are approximately seven times more likely to die by suicide compared to the general population [7]. However, 80% of individuals with eating disorders do not receive treatment [9]. Therefore, early intervention to link these individuals with specialized treatment is urgently needed.

In Japan, BN has become an important public health concern and is typically found among young women. A questionnaire survey for Japanese female students in 2002 (*N* = 3,031, average age ± SD = 17.2 ± 1.7) that attempted to assess their eating-related psychopathology and behaviors resulted in a point prevalence rate for BN in Japan of 2.32% (95% confidence interval, 1.79–2.86), a definite increase from that in 1982 [10]. Thus, BN is increasingly being recognized as a social problem in Japan.

Cognitive behavioral therapy (CBT) for patients with BN and BED has been shown to be effective and durable. Controlled studies of CBT have shown benefit not only for binge or purge frequencies but also for cognitive symptoms [11–13]. CBT is one of the most used evidence-based psychological interventions for BN, and clinical practice guidelines for eating disorders therefore generally recommend the use of CBT for patients with BN [14–16].

Unfortunately, there is a shortage of therapists appropriately trained in CBT in Japan to treat all patients who would benefit from this therapy. One of the origins of this dearth can be traced to the payment system for medical services for eating disorder treatments in Japan, which predominantly accommodates inpatient care, thereby neglecting the outpatient spectrum [17]. Given the extant landscape of the Japanese medical framework, despite the presence of a certain patient population, remuneration remains disproportionately low when juxtaposed with the temporal and cognitive investments necessitated for the treatment of eating disorders. Concurrently, a paucity of specialized facilities dedicated to the management of eating disorders further compounds this predicament [18, 19]. As a counteractive measure, earnest and concerted endeavors among the medical corps are needed to heighten the consciousness regarding eating disorders, while concurrently embarking upon the establishment of an amalgamative treatment paradigm that fosters collaborative synergies among a diverse array of medical establishments dispersed across the Japanese landscape. Furthermore, it is difficult for young women who have school activities or active social lives to make regular visits to a hospital or clinic, leading to a pattern of dropping out from continuing care. These issues make CBT relatively unavailable to many of those in need. Improved access to psychological care, including CBT, is very much needed in Japan.

On the other hand, the effectiveness of low-intensity CBT such as CBT-based self-help has been well documented [20, 21]. A meta-regression analysis has revealed that guided self-help (GSH) intervention is more effective in terms of adherence and treatment outcomes than “pure” self-help for BN [21]. Indeed, the National Institute for Health and Care Excellence (NICE) guideline recommended GSH with cognitive behavioral self-help materials for eating disorders as a first-line treatment for BN and CBT-ED as the next-line therapy [14].

Internet-based psychotherapy can overcome various barriers of face-to-face interventions because of its ease of accessibility, particularly for those with time limitations [22]. A controlled study by Fernández-Aranda et al. demonstrated that internet-based GSH with CBT concepts significantly improved the psychopathology and bulimic behavior of their participants compared to those on a waiting list [23]. However, there is as yet no available evidence-based iCBT in Japanese clinical settings.

This is the first study in Asia to examine adaption of the European-based Salut BN program. The main objective of this study was to demonstrate the feasibility and efficacy of iGSH-CBT for BN in Japan by assessing the change in binge eating frequency as a primary endpoint.

## Methods

This was an open-label feasibility study of patients with BN or BED; there was no control group. This study was first posted on April 1, 2018 on a clinical trial registry site (UMIN Clinical trials Registry Number: UMIN000031962). The study was approved by the Institutional Review Board of Chiba University Hospital on March 7, 2018 (G29054, No. 331) and the ethics committee of Sodegaura Satsukidai Hospital on April 6, 2019. The first patient was recruited on July 13, 2019.

### Participants

We recruited participants from July 2019 to April 2020. Nine female outpatients with BN (*n* = 5) or BED (*n* = 4) who were recruited from the outpatient unit of Chiba University Hospital and Sodegaura Satsukidai Hospital participated in this study. The participants met the current requirements for a diagnosis of BN and BED according to the Diagnostic and Statistical Manual of Mental Disorders, Fifth Edition (DSM-5) criteria [24] and had to be within the age range of 16–40 years.

The exclusion criteria were as follows: 1) organic brain complications/disorders, 2) substance use disorders, 3) patients highly likely to attempt suicide, 4) severe mental disorders requiring hospitalization, 5) severe medical illness, 6) severe social or occupational dysfunction or severe dysfunction in school associated with psychiatric symptoms, 7) currently receiving CBT, 8) patients for whom the program was considered unsafe for any other reason.

### Intervention: iGSH-CBT

**Fig. 1:**
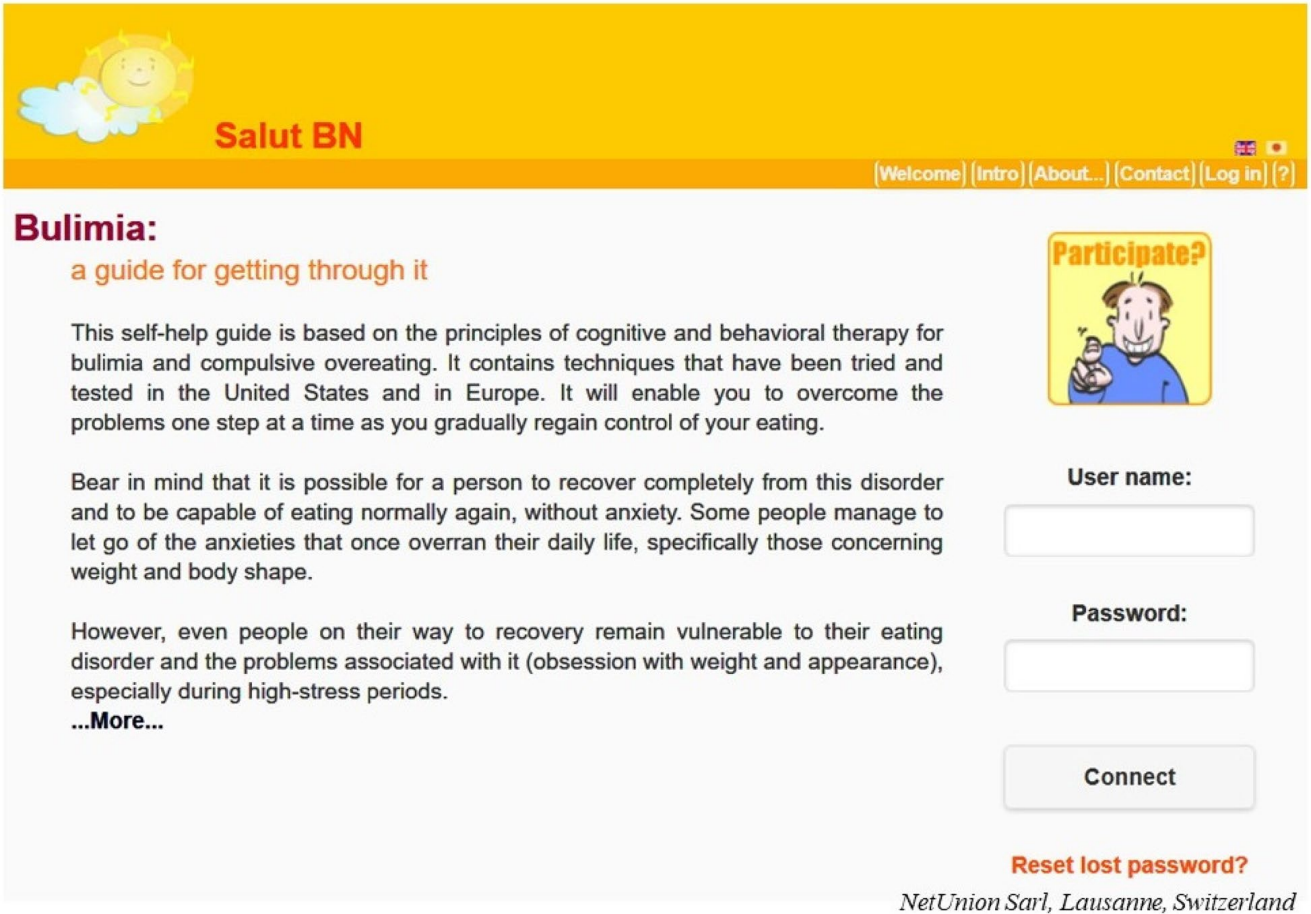
Login screen (Salut BN)

**Fig. 2:**
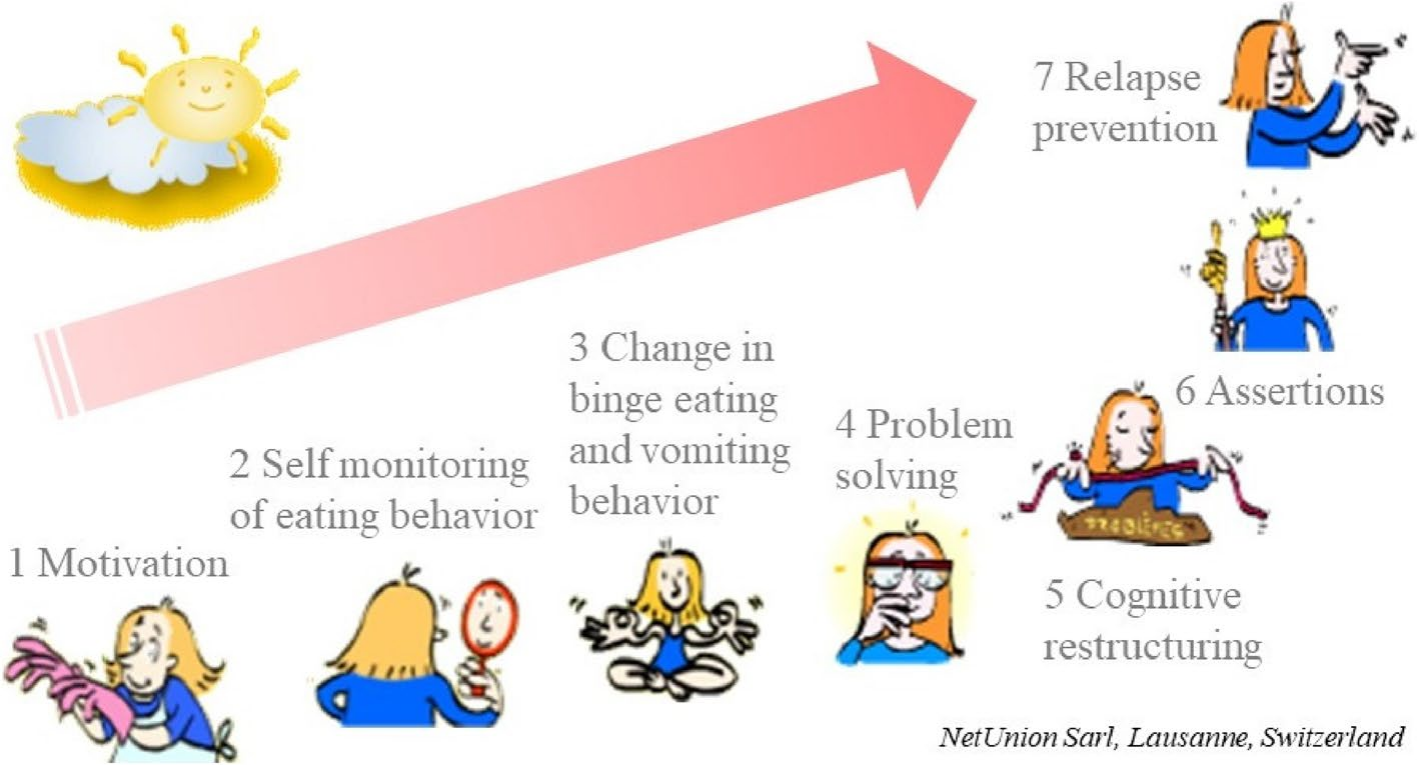
Seven STEPs (Salut BN)

For the iGSH-CBT program (Figs. 1 and 2), the authors (N.O., H.K., Y.H. and M.N.) adapted the Salut BN program for a Japanese audience and subsequently gained approval of the adaptations from the original author (Tony Lam, director of Net Union, a provider of health management software). The coaches in the program (N.O., H.K. and M.N.) received a 3-h lecture on coaching that was in compliance with the principles of Salut BN as laid out by the original author. Coaches provided weekly guidance to participants by email. Interventions were predominantly oriented toward expediting advancement through the program, while also addressing inquiries from participants pertaining to the program’s content. In addition, participants received regular psychiatric care during their 16-week iGSH-CBT program. Allocation of participants was predicated on a specific weekday under a coach’s supervision in addition to the designated facility.

### Clinical measurements

The patients were evaluated at pre-treatment (T0), at the end of the 16-week treatment (T1), and at 24 weeks as follow-up after the treatment (T2). The primary outcome evaluated was the average change in weekly frequency of objective binging between pre- and post-16-week treatment.

Secondary outcomes were changes in the weekly frequencies of objective purge episodes and monthly objective binging, the dropout rate, self-report questionnaires of the frequencies of binging and purging, psychopathological characteristics of eating disorders according to the Bulimia Investigatory Test, Edinburgh (BITE) [25], Eating Disorder Examination Questionnaire (EDE-Q) [26], Eating Disorder Inventory-2 (EDI-2) [27], Hospital Anxiety and Depression Scale (HADS) [28], the 5-level Euro Qol 5 dimension (EQ-5D) [29, 30], measurements of motivation, and the intervention completion rate. In addition, Autism-Spectrum Quotient (AQ) [31] and Adult ADHD Self-Report Scale (ASRS) [32] were used to assess developmental disabilities at pre-treatment (T0). All evaluation scales were validated for operation in Japanese [33–37].

The patients were evaluated at baseline (T0), at the end of the 16-week intervention (T1), and at 24 weeks (follow-up, (T2)). All evaluations were conducted face-to-face. To reliably perform these evaluations, physicians participating in the study underwent several rounds of assessment training. The physicians conducted a structured interview at the baseline visit and made an assessment regarding current conditions and comorbid psychiatric disorders. AQ and ASRS were used to assist in the assessment of autism spectrum disorders (ASD) and attention-deficit hyperactivity disorder (ADHD).

### Statistical analysis

This was a two-center study (Chiba University Hospital and Sodegaura Satsukidai Hospital), and considering the number of BN and BED patients per year at these facilities, a sample size of 20 was considered appropriate in terms of feasibility. The objectives of this study were to assess the feasibility (i.e., system usability, treatment adherence, acceptability of the program in the Japanese language and culture) of the program, to estimate effect sizes for the purpose of designing a larger study, and to test the acceptability of iGSH-CBT in Japanese clinical settings. From our estimation of 15 outpatients with BN per year and considering drop-out rates of 30–40% according to previous studies, our resulting estimation arrived at 9 patients.

The primary and secondary analyses were based on the full analysis set, which was defined as all participants with efficacy data who were enrolled in the clinical study and who experienced the iGSH-CBT program at least once. A per-protocol analysis that included all patients in the full analysis set but excluded those who met any of the significant deviations from the study procedure, such as inclusion/ exclusion criteria not met, receiving prohibited concomitant drugs or therapies, or non-complying with the study program, was not conducted because the subject group was the same as the full analysis set. Paired-t test was used for comparison between changes within each subject.

Data were analyzed with SAS statistical software (version 9.4, SAS Institute, Cary, NC, USA) and R version 4.0.5 (R Foundation for Statistical Computing, Vienna, Austria.).

## Results

### Participants

**Fig. 3:**
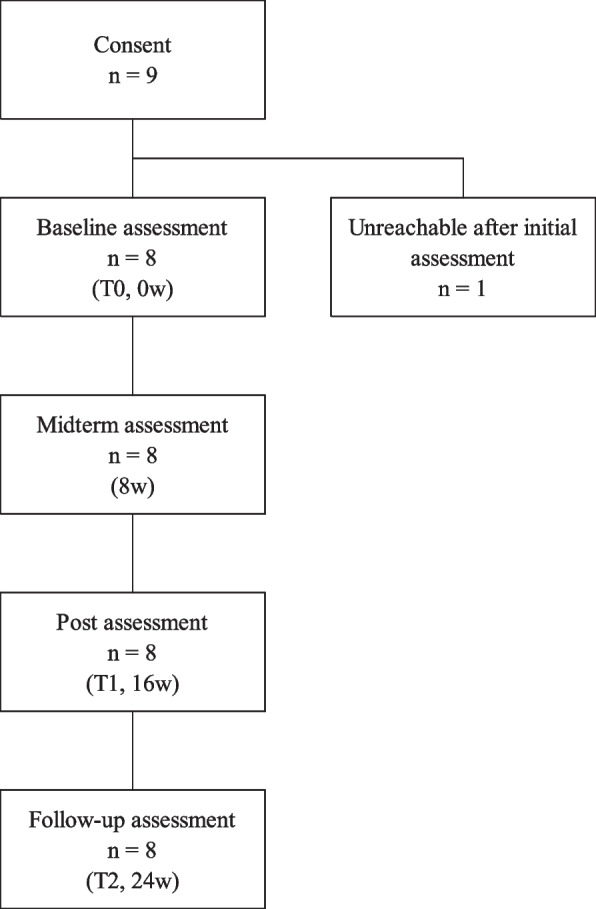
Study flow

Eight female participants received baseline assessment and were confirmed to have never previously received CBT or GSH. Figure 3 shows that one participant (11.1%) was unreachable after the initial assessment while the others (*n* = 8, 88.9%) underwent all assessments: baseline, midterm, post-treatment and follow-up assessment. The reason one participant did not attend the clinic is unknown. No adverse events occurred.

### Baseline characteristics

All participants were female with a mean age of 28.0 years (range 16–38, SD = 8.0). Equal numbers of participants were diagnosed with BN and BED. Fifty percent were diagnosed with mood (affective) disorders (F3, ICD-10) as comorbid psychiatric diagnoses and 37.5% (*n* = 3) had a tendency toward ADHD. Mean BMI was 22.2 (range 19.7‒27.7; SD = 2.8) (Table 1).

**Table 1:** Patient characteristics

	*n* = 8
Age, years	
Mean (SD)	28.0 (8.0)
Female, n (%)	8.0 (100.0%)
Period of ED, years	
Mean (SD)	4.5 (5.1)
Diagnosis, n (%)	
BN	4.0 (50.0%)
BED	4.0 (50.0%)
AQ score	
Mean (SD)	25.2 (5.8)
ASRS score	
Mean (SD)	12.8 (4.5)
BMI	
Mean (SD)	22.2 (2.8)
Minimum BMI	
Mean (SD)	16.4 (2.3)
Minimum BMI, age, years	
Mean (SD)	20.0 (5.9)
Maximum BMI	
Mean (SD)	24.9 (3.6)
Maximum BMI, age, years	
Mean (SD)	24.1 (7.4)
Years of education	
Mean (SD)	13.4 (2.4)

*Abbreviations*: *SD* Standard deviation, *ED* Eating disorder, *BN* Bulimia nervosa, *BED* Binge eating disorder, *BMI* Body mass index

### Treatment outcomes

The weekly frequency of objective binge eating, which serves as the principal outcome measure, exhibited values of 6.90 times per week pre-treatment (T0), 5.40 times per week post-treatment (T1), and 2.80 times per week at follow-up (T2). There was no significant difference in the frequency of overeating between pre- (T0) and post- (T1) treatment, the mean rate of change being -4.40% (*p* = 0.914). At the end of the treatment, 25% (*n* = 2) had no bulimic symptoms and 75% (*n* = 6) had decreased their frequency of binging by 25% or more (Table 2).

**Table 2:** Comparison of changes: pre-, post-treatment, and follow-up

	Pre-treatment (T0) Mean (SD)	Post-treatment (T1) Mean (SD)				Follow-up (T2) Mean (SD)			
			Comparison (pre- vs. post-)				Comparison (pre- vs. follow-up)		
			*P*-value	Mean changes	CI 95%		*P*-value	Mean changes	CI 95%
Binge eating frequency (1w)	6.90 (6.00)	5.40 (4.80)	0.396	1.5	(-2.4; 5.4)	2.80 (3.00)	0.058	4.1	(-0.2; 8.4)
Binge eating frequency (4w, EDE-Q)	24.00 (15.00)	19.00 (19.00)	0.329	5.2	(-6.6; 17.1)	12.00 (9.00)	0.011**	12.2	(3.8; 20.7)
Self-induced vomiting frequency (4w, EDE-Q)	10.00 (17.00)	12.00 (22.00)	0.474	-2.0	(-8.3; 4.3)	11.00 (20.00)	0.673	-1.0	(-6.4; 4.4)
EDE-Q Restraint	2.42 (1.48)	1.68 (0.78)	0.163	0.8	(-0.4; 1.9)	1.98 (0.80)	0.473	0.4	(-1.0; 1.9)
EDE-Q Eating Concern	4.15 (1.98)	3.40 (1.46)	0.259	0.8	(-0.7; 2.2)	2.50 (1.36)	0.473	0.4	(-1.0; 1.9)
EDE-Q Weight Concern	4.92 (0.80)	4.12 (1.10)	0.084	0.8	(-0.1; 1.7)	3.05 (1.08)	0.004**	1.9	(0.8; 2.9)
EDE-Q Shape Concern	5.11 (0.83)	4.53 (1.04)	0.112	0.6	(-0.2; 1.3)	3.44 (1.26)	0.011**	1.7	(0.5; 2.8)
EDE-Q Global score	4.15 (0.78)	3.43 (0.81)	0.119	0.7	(-0.2; 1.7)	2.74 (0.90)	0.016**	1.4	(0.4; 2.5)
EDI-2	109.00 (24.00)	108.00 (35.00)	0.888	1.4	(-20.9; 23.7)	86.00 (28.00)	0.038**	23.5	(1.6; 45.4)
BITE-SAS	15.10 (6.10)	17.00 (7.40)	0.414	-1.9	(-7.0; 3.2)	13.60 (8.30)	0.233	1.5	(-1.2; 4.2)
BITE-SS	12.00 (8.00)	11.00 (7.00)	0.646	1.0	(-3.9; 5.9)	9.00 (5.00)	0.201	2.9	(-1.9; 7.7)
HADS total	23.40 (3.20)	19.90 (3.40)	0.026**	3.5	(0.6; 6.4)	21.10 (4.90)	0.214	2.2	(-1.6; 6.1)
EQ-5D-5L	0.78 (0.16)	0.81 (0.14)	0.519	0.0	(-0.1; 0.1)	0.81 (0.19)	0.756	0.0	(-0.2; 0.2)

*Abbreviations*: *SD* Standard deviation, *CI* Confidence interval, *BITE-SAS* Symptom scale, *BITE-SS* Severity scale, *BMI* Body mass index, *ASRS-ITS* Inattention Trait Score, *ASRS-HTS* Hyperactivity Trait Score

^*^Paired T test

^**^Bold: significant comparison

Table 2 shows various pre- (T0) and post-treatment (T1) values and those at follow-up (T2). In the comparison between T0 and T1, HADS total scores were significantly improved (*p* = 0.026). In the comparison between T0 and T2, the EDE-Q measure of binge eating frequency in the past 4 weeks (*p* = 0.011), EDE-Q weight concern (*p* = 0.004), EDE-Q shape concern (*p* = 0.011), EDE-Q global score (*p* = 0.016) and EDI-2 score (*p* = 0.038) had all significantly improved. There were no improvements in BITE or EQ-5D-5L. At the end of the 16-week intervention, the drop-out rate was 11.1%.

### Comparison with previous studies

Table 3 shows a comparison of the results of previous and current studies of Salut BN programs for BN participants. In the present study, the drop-out rate of 11.1% at the end of the 16-week intervention was lower than those of previous studies. In addition, 25% of the participants exhibited an absence of binging symptoms, which mirrors what other studies have found. The mean age of the participants was 28 years, slightly higher than the preceding age averages. The present study uniquely focuses on participants diagnosed with BED.

**Table 3:** Comparison of SALUT-BN project studies

**Study**	**Participants**				**Control group**	**Intervention period**	**Post-treatment**			
	**n**	**diagnosis**	**age**	**(SD)**			**no binging**		**drop-out**	
							**iGSH-CBT**	**control**	**iGSH-CBT**	**control**
This study	8	BN BED	28.0	(8.0)	n/a	4 M	25.0%	n/a	11.1%	n/a
F Fernández-Aranda et al., 2009 [23]	62	BN	23.7	(3.6)	WL	4 M	32.3%	3.2%	45.0%	n/a
I Carrard et al., 2011 [38]	127	BN EDNOS	24.7	(5.1)	n/a	4 M	23.0%	n/a	25.2%	n/a
G Wagner et al., 2014 [39]^a^	126	BN EDNOS	iGSH-CBT 24.17 control 25.02	(4.46) (3.84)	guided bibliotherapy	4–7 M	48.6%^b^	58.3%^b^	37.1%	32.1%

^a^Study on predictors and drop-outs for the efficacy of iGSH-CBT

^b^Remission: does not fulfill DSM-IV criteria

## Discussion

This pilot intervention study was conducted to examine the feasibility of iGSH-CBT for BN or BED in a Japanese clinical setting as a prelude to a randomized controlled trial (RCT) and to evaluate its feasibility and acceptability to an Asian audience. Notably, no incidents were encountered over the course of either the treatment phase and subsequent monitoring, and the deployment and execution of the program transpired without any significant challenges, thereby affirming the practicability of iGSH-CBT for Japanese patients with BN and BED. Regarding the primary outcome, the weekly frequency of objective binge eating, the intervention was estimated to have had a statistically insignificant effect at the end of the 16-week treatment period (T1) and at 24 the week follow-up (T2). Conversely, in relation to the monthly (the last 4 weeks) frequency of objective binge eating, a significant decline was observed at the 24 week follow-up (T2) (*P* < 0.05, CI 3.8‒20.7), but not at the end of the 16-week period (T1). As for the divergence among these findings, one possible reason is the limitation due to the small number of participants. Although a majority of participants displayed a decrease in binge eating frequency, it was also observed that a small number of participants revealed a large increase in binge eating frequency. Regardless, we consider that this, at the very least, implies the potential for the effects of the 16-week treatment regimen to endure for a duration subsequent to the program’s culmination. Furthermore, at the end of the 16-week intervention (T1) 25% of the participants had achieved abstinence, and no severe adverse events were observed over the course of treatment. As shown in Table 3, previous studies on Salut BN among patients of similar age and clinical background achieved abstinence from 23.0 to 48.6% [23, 38, 40]. Our result fell within that range. During the trial, participants who achieved abstinence responded more frequently to their coaches using the messaging tool in Salut BN than did the other participants, demonstrating their high motivation for treatment. This is consistent with the results of a previous study that indicated that high motivation is one of the predictors of good prognosis in iGSH-CBT [39], but validation with a larger number of participants will still be required.

As for the results of self-report questionnaires, the secondary outcome, the HADS total score at the end of the 16-week intervention (T1), and the EDE-Q subitems (weight concern, shape concern, and global score) and EDI-Q at 24 weeks as follow-up after the treatment (T2) demonstrated superior improvement. This finding suggests another possibility, namely, that the impacts of the iGSH-CBT program might manifest themselves belatedly. Two prior studies showed improvement in eating disorder severity with respect to EDI-Q at the end of treatment, but were not evaluated with respect to follow-up duration [23, 38]. Therefore, this hypothesis needs to be verified in future prospective, larger studies.

A meta-analysis of ninety-nine RCTs by Linardon et al. showed an overall drop-out rate from CBT of 24% (95% CI = 22–27%), whereas the drop-out rate from internet-based CBT was 33% (95% CI = 25–34%) [41]. At the end of the 16-week intervention in this study, the drop-out rate was 11.1%, which was less than those of previous studies with the Salut BN programs for BN participants (Table 3).

This pilot program is based on a CBT self-help manual that is distinctive in two respects. First, it consists of 7 modules with concepts, examples and exercises: 1) motivation, 2) self-observation, 3) modification of behavior, 4) observation and modification of automatic thoughts, 5) problem solving, 6) self-affirmation, 7) conclusion and relapse prevention [23, 42]. One of these exercises is a “daily food diary”, which takes approximately 5 to 10 min per day to fill in. Patients manage the time that they can and want to spend using the program, and they can work through the program at their own speed. Second, patients are required to take part in a weekly email support program with their trained counselors throughout the intervention period. Thus, patients can receive treatment without being restricted to regular visits to a hospital or clinic, which may increase the accessibility and the availability of CBT. While differences in treatment effects due to different coaches should also be examined, these differences are unlikely to exert a substantial effect, as participants are exclusively dedicated to adhering to the prescribed regimen and the coach has received sufficient time for training.

Although we modified food guides according to food availability and the culinary culture and eating habits in Japan, we found that no culture-related modifications of the treatment based on the CBT self-help manual were required for Japanese patients (https://www.fao.org/nutrition/education/food-dietary-guidelines/home/en/).

There are several methodological limitations, primarily because this was a small-scale, open-label, single-arm feasibility study. Strong inferences cannot be made about the efficacy of iGSH-CBT due to the small sample size. Second, although the Salut BN program was developed for patients with BN, we assessed patients with BN and BED in this study. The three articles delineated in Table 3 encompassed psychiatric diagnoses grounded in DSM-4 [43]. However, within two of the references, eating disorder not otherwise specified (EDNOS) was included concomitantly with BN. Within the 2013 iteration of the DSM-5, the diagnostic criteria underwent partial revision, leading to the establishment of BED as a new independent diagnostic criterion. Consequently, the inclusion of BED within this study was deemed reasonable. A previous systematic review demonstrated moderate support for the efficacy of CBT and GSH for BED [13]. However, our data suggest that further research such as an RCT is warranted to investigate the applicability of this therapeutical tool to patients with BN and BED in Japan.

Considering that many individuals with BN and BED do not have access to specialized treatment, multiple evidence-based treatment options need to be made available. The present iGSH-CBT intervention, an adaptation of the Salut BN program developed for European audiences, has the potential to link such individuals with appropriate healthcare in Japan. This study provided preliminary evidence that iGSH-CBT is feasible among Japanese patients who have BN or BED. The dropout rate was low, and no severe adverse events were observed over the course of the treatment.

## Conclusions

Overall, our study findings indicate that iGSH-CBT is an acceptable and possibly even a preferred method of CBT delivery for Japanese patients with BN or BED and that our Japanese adaptation of Salut BN is feasible. Further study using an RCT is warranted.

## Data Availability

Data supporting the findings of this study are available from the Chiba University Clinical Research Center, although restrictions apply to their availability. These data were used under license for the current study, and so are not publicly available. Data are, however, available from the authors upon reasonable request and with permission of the Chiba University Clinical Research Center.

## Abbreviations

iGSH: Internet-based Guided Self-Help
CBT: Cognitive behavioral therapy
BN: Bulimia nervosa
BED: Binge eating disorder
ED: Eating disorder
DSM: Diagnostic and Statistical Manual of Mental Disorders
BITE: Bulimic Investigatory Test, Edinburgh
EDE-Q: Eating Disorders Examination Questionnaire
EDI-2: Eating Disorder Inventory-2
HADS: Hospital Anxiety and Depression Scale
EQ-5D: 5-Level Euro QOL 5 dimension
RCT: Randomized controlled trial
